# A subglottic foreign body mimicking croup: A case report

**DOI:** 10.1097/MD.0000000000026609

**Published:** 2021-07-16

**Authors:** Hong Chan Kim, Chung Man Sung, Hyung Chae Yang

**Affiliations:** a Department of Otolaryngology–Head and Neck Surgery, Chonnam National University Hwasun Hospital and Chonnam National University Medical School, Hwasun; b Department of Otolaryngology–Head and Neck Surgery, Chonnam National University Hospital and Chonnam National University Medical School, Gwangju, South Korea.

**Keywords:** croup, foreign body aspiration, laryngeal foreign body, stridor

## Abstract

**Introduction:**

Foreign body (FB) aspiration is one of the causes of respiratory distress in infants is an extremely dangerous and potentially life-threatening event. The diagnosis of FB aspiration is difficult because the signs and symptoms vary according to the degree of airway blockage or location of the FB.

**Patient concerns:**

An 11-month-old female infant visited a hospital because of a sudden onset cough. She was relatively healthy without fever, rhinorrhea cyanosis, or poor feeding. On physical examination, auscultation revealed inspiratory stridor without wheezing and crackles.

**Diagnosis:**

Croup was suspected when considering the history, physical examination, and imaging. However, she did not respond to a 4-day course of treatment for croup. Flexible laryngoscopic examination was performed, and we identified a thin, flat, and sharp FB embedded in the subglottic region.

**Interventions:**

Emergency surgery was performed to remove the FB. Short-term intravenous corticosteroids and antibiotics were used to prevent laryngeal swelling and aspiration pneumonia.

**Outcomes:**

One week after the procedure, the laryngeal mucosa had completely healed.

**Conclusion:**

FB aspiration should be considered in an infant with an impression of croup. In particular, if there is no response to medical or conservative treatment for croup, further evaluation is needed.

## 1 Introduction

Infants with dyspnea may encounter a catastrophic situation if the diagnosis is incorrect or delayed. A doctor must be familiar with the pathophysiological features, signs, and common causes of dyspnea in infants to minimize misdiagnosis. A comprehensive history and physical examination are crucial for evaluating infants with dyspnea because they cannot be reported by the patient. Sudden or progressive onset, previous dyspnea event, history of respiratory infection, episode of foreign body (FB) swallowing, symptoms, or auscultation are important clues for diagnosing the condition.^[1]^

Herein, we report a rare case of an 11-month-old infant with subglottic FB masquerading as croup. We think that our case provides excellent images for a subglottic FB and is educationally valuable.

## 2 Case report

An 11-month-old female infant visited a local doctor because of a sudden onset cough. She was relatively healthy without fever, rhinorrhea cyanosis, or poor feeding. Her mother did not witness any particular events at home. On physical examination by the local doctor, auscultation revealed inspiratory stridor without wheezing and crackles. With the impression of mild croup, she was prescribed an antitussive drug and dexamethasone. Her caregivers were educated about supportive care, including humidification and hydration. However, the inspiratory stridor persisted without improvement when she visited a local doctor again after 4 days, and she was referred to our emergency room to determine the cause on October 22, 2020.

**Figure 1:**
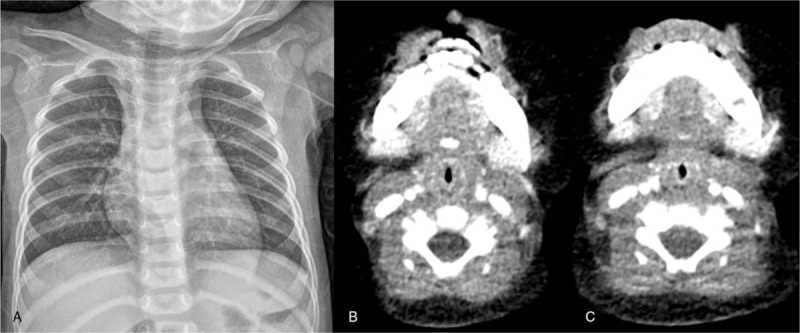
Imaging findings. Imaging studies show no active consolidation or mass in either lung, no abscess formation in the neck, and no detectable foreign body in the tracheobronchial tract. A: Anteroposterior chest view. B: Vocal cord level, C: Subglottic level.

**Figure 2:**
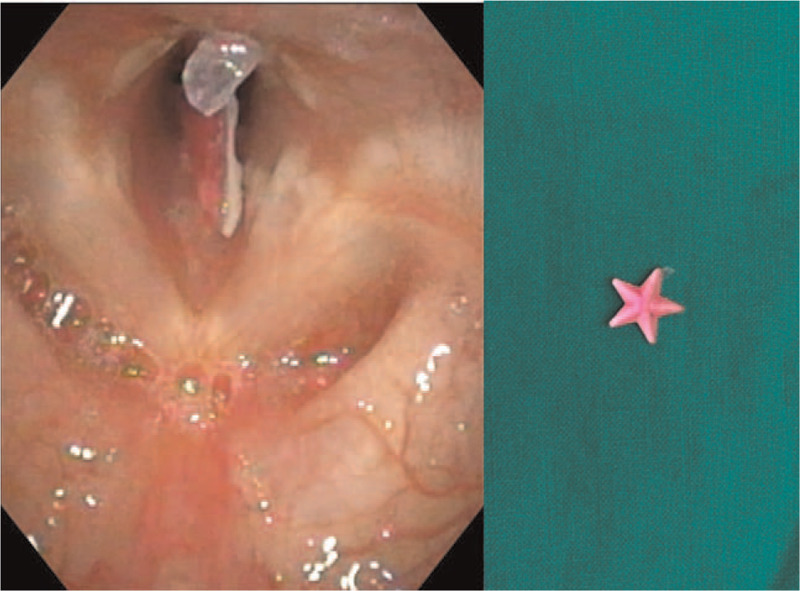
Flexible laryngoscopy findings. Flexible laryngoscopy demonstrates a star-shaped foreign body caught on the subglottis with relatively airway patency.

She was not febrile, with stable vital signs. On physical examination, the patient exhibited inspiratory stridor without wheezing and crackles. Laboratory studies showed an elevated white blood cell count (14.2 × 10^3^/μL) with normal plasma C-reactive protein (0.03 mg/dL). We performed chest radiography and computed tomography (CT) but could not identify any abnormal findings (Fig. 1). Flexible laryngoscopic examination was performed to confirm the larynx anatomy. We identified a thin, flat, and sharp FB embedded in the subglottic region (Fig. 2) (Supplemental Video).

**Figure 3:**
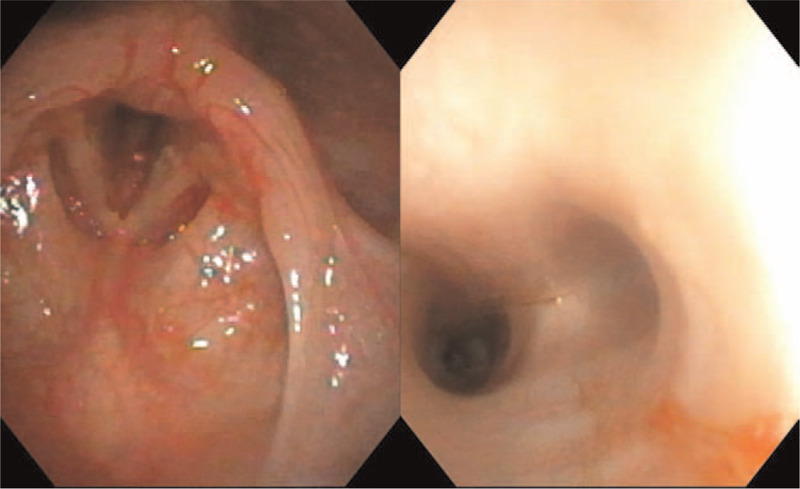
Postoperative bronchoscopy findings. On the bronchoscopy performed immediately after removing the foreign body (FB), there is laryngeal mucosa tearing with a little bleeding but no granulation tissue or remnant FB in the trachea.

Emergency surgery was performed under general anesthesia. Oxygen saturation was maintained through mask bagging because of the inability to perform intubation. When oxygen saturation was sufficiently elevated, the FB was removed by direct laryngoscopy after mask bagging was stopped. Bronchoscopy was performed immediately after removing the FB; there was laryngeal mucosa tearing with a little bleeding but no granulation tissue or remnant FB in the trachea (Fig. 3). Short-term intravenous corticosteroids and antibiotics were used to prevent laryngeal swelling and aspiration pneumonia. One week after the procedure, the laryngeal mucosa had completely healed, as determined by flexible laryngoscopy.

## 3 Discussion

FB aspiration is one of the causes of respiratory distress in infants is an extremely dangerous and potentially life-threatening event. The diagnosis of FB aspiration is difficult because the signs and symptoms vary according to the degree of airway blockage or location of the FB. The most common sites of FB aspiration in children are as follows: the right bronchi (60%), left bronchi (23%), trachea/carina (13%), larynx (3%), and bilateral (2%).^[2]^ Generally, the longer an FB remains in the tracheobronchial system, the more serious the complication.

Common signs of bronchial FB are coughing, acute onset of choking, and wheezing. If these patients have a delayed diagnosis, they may present with fever and symptoms of pneumonia. Laryngeal FB often manifests as stridor, unlike bronchial FB. The patient may also present with pain and difficulty with speaking and breathing.^[3]^ Therefore, FB aspiration must be differentiated from croup, which is a common cause of acute upper respiratory symptoms, including inspiratory stridor, hoarse voice, and coughing in infants.^[4]^

Chest radiography is the most commonly used tool in the initial assessment of FB aspiration. However, the presence of normal findings on chest radiography should not rule out FB aspiration. Indeed, up to 50% of cases can have normal findings on radiography.^[5]^ CT has been proposed as a more sensitive modality than conventional radiography. However, it is difficult to identify very small, thin, and radiolucent FBs on CT.^[6]^ Flexible laryngoscopy is a very useful diagnostic method because it is rapid and the FB is clearly visualized. Nevertheless, if children have poor cooperation, it is not only difficult to perform, but may also provide them with a terrible experience. Rigid bronchoscopy is the gold standard for the diagnosis or treatment of FB aspiration in children. Therefore, it should be performed in all cases of suspected FB aspiration. It permits control of the airway, good visualization, manipulation of the FB with a variety of forceps, and immediate management of mucosal hemorrhage.^[7]^ However, it is recommended that an experienced operator perform FB removal to minimize the risk of complications such as pneumothorax, remnant FB, and respiratory arrest.

In our patient, croup was suspected when considering the history, physical examination, and imaging. However, she did not respond to a 4-day course of treatment for croup. Our patient's FB was lodged in the subglottis without passing through the trachea because of its large size, which induced stridor. In addition, it was thin and flat; thus, there were no symptoms other than cough, and the FB was not detected on chest X-ray or CT. Like this, subglottic FB aspiration with partial airway obstruction could present similar to croup, the symptoms of which include cough, hoarse voice, dyspnea, and inspiratory stridor.

In conclusion, FB aspiration should be considered in an infant with an impression of croup. In infants with these symptoms, a detailed history should be taken and a physical examination performed, even if the caregiver did not witness FB aspiration. In particular, if there is no response to medical or conservative treatment, further evaluation is needed. If there are doubts about FB aspiration, physicians should not hesitate to perform rigid bronchoscopy or flexible laryngoscopy.

## Acknowledgments

The authors would like to thank Editage (www.editage.com) for English language editing.

## Author contributions

**Investigation:** Chung Man Sung.

**Supervision:** Hyung Chae Yang.

**Visualization:** Chung Man Sung.

**Writing – original draft:** Hong Chan Kim.

**Writing – review & editing:** Hyung Chae Yang

## Supplementary Material

Supplemental Digital Content
